# Development and psychometric validation of the short-form mandarin Chinese demoralization scale for cancer patients

**DOI:** 10.3389/fpsyg.2026.1834425

**Published:** 2026-06-16

**Authors:** Yapeng Wang, Cangmei Fu, Xiaoxin Liu, Min Yao, Guangze Zhao

**Affiliations:** 1 Department of Oncology, Guilin Hospital of the Second Xiangya Hospital, Central South University, Guilin, Guangxi, China; 2 Department of Oncology, The Second Xiangya Hospital, Central South University, Changsha, Hunan, China; 3 Clinic Nursing Teaching and Research Section, The Second Xiangya Hospital, Central South University, Changsha, Hunan, China; 4 Xiangya Nursing School, Central South University, Changsha, Hunan, China

**Keywords:** cancer, demoralization, psychometric, Rasch model, reliability, scale refinement, validity

## Abstract

**Background:**

Demoralization is a common psychological distress in Chinese cancer patients, affecting treatment adherence and quality of life. NCCN Guidelines recommend routine monitoring of distress. The original 24-item Mandarin Demoralization Scale (DS-MV) is comprehensive but less suited for rapid, repeated screening in busy oncology clinics. We therefore refined and validated a short-form Mandarin Demoralization Scale (s-DS-MV) for efficient demoralization assessment.

**Methods:**

This psychometric validation study initially enrolled 1,050 Chinese cancer patients between June 14, 2022 and June 13, 2023. Of these participants, 971 completed the original 24-item DS-MV through face-to-face questionnaire administration. Exploratory factor analysis and Rasch modeling were used to evaluate, refine, and revalidate the scale, including examinations of dimensionality, response scale appropriateness, item fit, item bias, and item difficulty. Test–retest reliability was assessed in a subgroup of 50 patients with relatively stable clinical symptoms.

**Results:**

The revised 14-item s-DS-MV (3-point Likert format) showed a 2-subscale structure supported by exploratory factor analysis. Rasch modeling confirmed acceptable model fit, unidimensionality and compliance with core criteria for each subscale. The scale exhibited satisfactory internal consistency and test–retest reliability (Existential Emptiness and Affective Distress subscale: 9 items, *α* = 0.855, intraclass correlation coefficient (ICC) = 0.934; Self-Worth and Life Valuation subscale: 5 items, *α* = 0.708, ICC = 0.881; full scale: α = 0.864, ICC = 0.933).

**Conclusion:**

The s-DS-MV is a 3-point two-factor self-report scale with robust psychometric properties and favorable screening utility, making it well-suited for rapid clinical screening, dynamic monitoring, and related research on demoralization among cancer patients with similar demographic and clinical characteristics in Hunan and adjacent regions of China.

## Introduction

Cancer remains a pressing global public health challenge, with its burden steadily increasing and posing substantial threats to population health worldwide. According to the Global Burden of Disease Study 2023, 18.5 million new cancer cases and 10.4 million cancer deaths occurred globally in 2023, excluding non-melanoma skin cancers (GBD 2023 Cancer Collaborators, 2025). Projections indicate that by 2050, cancer incidence and mortality will rise by 60.7 and 74.5%, respectively, compared with 2024 levels, with a disproportionately sharp increase expected in low- and middle-income countries (GBD 2023 Cancer Collaborators, 2025). Beyond its substantial epidemiological and demographic effects, cancer also places multidimensional distress on patients, spanning physical, psychological, social, and spiritual domains (Li et al., 2025; Liu et al., 2024; NCCN, 2026). Such distress can undermine patients’ ability to effectively cope with the disease and its physical symptoms, while also reducing the efficacy of anti-neoplastic treatments (Fang et al., 2014; Li et al., 2025; Liu et al., 2024; Philipp et al., 2025; Wu et al., 2021; Zeng et al., 2024; Zheng et al., 2022). Distress in patients with cancer exists on a continuous spectrum, ranging from common emotional experiences such as vulnerability, sadness, and fear to severe, functionally limiting conditions including depression, anxiety, social isolation, existential crisis (including demoralization), and spiritual crisis (Li et al., 2025; NCCN, 2026).

Demoralization, a prevalent psychiatric condition increasingly recognized in cancer populations, is officially classified as a distinct psychological entity endowed with clear clinical specificity in the 11th Revision of the International Classification of Diseases (ICD-11; Code MB22.2) — defined by a loss of confidence in one’s coping capacity, accompanied by core feelings of helplessness, hopelessness and discouragement, and encompassing a broader negative mental state characterized by dysphoria, disheartenment, loss of meaning and purpose, and a sense of failure (Hua and Bhatarasakoon, 2025; Zheng et al., 2022). Its emergence is closely associated with multiple stressors experienced by cancer patients, including persistent symptom burden, disruptions to social roles, low social support, coping difficulties, and other forms of emotional distress (Fang et al., 2014; Gan et al., 2022; Hong et al., 2022; Hua and Bhatarasakoon, 2025; Li et al., 2025; Peng et al., 2021; Robinson et al., 2015). Epidemiological evidence indicates a prevalence range of 16.0–57.6% among cancer patients, with a mean of 35.8% (Gan et al., 2022), which underscores the pervasive clinical impact of demoralization on this vulnerable population. Notably, this multifaceted negative psychological state not only inflicts severe emotional distress on patients but also contributes to a series of adverse clinical outcomes, including impaired quality of life, exacerbated depression and anxiety as well as suicidal ideation and a desire to die (Costanza et al., 2022; Fang et al., 2014; Hua and Bhatarasakoon, 2025). This critical association establishes it as a core high-risk indicator in cancer distress management and serves as a key assessment criterion for determining whether patients require referral to mental health teams in oncology settings (Costanza et al., 2022; NCCN, 2026). Consequently, timely identification, dynamic monitoring, standardized documentation and targeted intervention for demoralization are also recommended for incorporation into clinical practice across all clinical settings and the entire disease trajectory of cancer care, and carry profound clinical significance (Costanza et al., 2022; Hua and Bhatarasakoon, 2025; NCCN, 2026; Robinson et al., 2015).

Existing research demonstrates that, among screening tools utilized in demoralization-focused studies worldwide (Hua and Bhatarasakoon, 2025), half of these tools are disease-specific assessment instruments: the Demoralization Scale (DS, 24-item version; Kissane et al., 2004), accounting for 54%; the Demoralization Scale-II (16-item version; Robinson et al., 2016), with a proportion of 14%; and the Diagnostic Criteria for Psychosomatic Research (DCPR) (Rafanelli et al., 2005), representing 12%. Other relevant studies, by contrast, have resorted to a heterogeneous array of alternative assessment tools (Hua and Bhatarasakoon, 2025), including the Psychiatric Epidemiology Research Interview (PERI), Subjective Incompetence Scale (SIS), Beck Depression Inventory (BDI), Dysfunctional Attitudes Scale (DAS), Composite International Diagnostic Interview (CIDI), Brief COPE Scale, Calgary Depression Scale for Schizophrenia (CDSS), Short Demoralization Scale (SDS), Demoralization Interview (DI), Hopelessness Scale (HS), and Demoralization and Subjective Incompetence Scale (DSIS). In contrast, in Chinese clinical and research settings, only two such tools — the Demoralization Scale (DS) (Kissane et al., 2004) and the Demoralization Scale-II (DS-II) (Robinson et al., 2016) — have thus far undergone Chinese translation and cultural adaptation, and been formally applied in demoralization research. Among these, the Mandarin version of the Demoralization Scale (DS-MV) has emerged as the predominant assessment instrument, having demonstrated satisfactory psychometric properties across diverse cancer patient populations while also effectively differentiating demoralization from depression (Lin et al., 2022; Tang et al., 2020). Yet, despite its well-established psychometric properties, the 24-item DS-MV poses notable practical barriers in routine clinical practice: time constraints and patients’ profound fatigue and diminished functional capacity (common in patients with advanced illness) often impair its feasibility for broad implementation or repeated assessment — let alone dynamic self-reporting. To address this urgent unmet clinical need for a concise yet psychometrically robust assessment instrument, the present study aims to develop a short-form Mandarin Demoralization Scale (s-DS-MV) based on local Chinese cancer patient samples.

However, traditional psychometric evaluation based on classical test theory (CTT) yields limited information regarding item performance, person–item interactions, and response category functioning — all essential for rigorous, evidence-based scale refinement. As an advanced analytical approach, item response theory (IRT), particularly the one-parameter Rasch model, effectively addresses these limitations. The Rasch model enables comprehensive evaluation of unidimensionality, item fit, response threshold ordering, person-item targeting, differential item functioning, and local item dependency (DeVellis, 1991; Park and Park, 2019). This supports systematic item reduction while retaining the original scale’s core measurement structure. By integrating CTT and Rasch analyses, this study aimed to develop and validate a shortened version of the 24-item Mandarin Chinese Demoralization Scale (DS-MV), namely the s-DS-MV. The brief scale was optimized for clinical applicability and sound psychometric properties, targeting advanced cancer patients with demographic and clinical characteristics comparable to the regional sample from Hunan Province.

## Materials and methods

### Design and sampling

This cross-sectional study employed convenience sampling and adhered to the STROBE guidelines (von Elm et al., 2007). Eligible cancer patients were recruited from the oncology departments of two tertiary general hospitals in Changsha, Hunan Province, between June 14, 2022 and June 13, 2023. Recruitment was conducted by trained research staff who also served as ward nursing team leaders, with all enrollment completed during routine working hours.

The inclusion criteria were as follows: (a) histopathologically confirmed primary solid tumors staged II–IV per the Tumor-Node-Metastasis (TNM) system; (b) full awareness of personal cancer diagnosis; (c) age 18 years or older; (d) intact verbal and written communication capacity; (e) voluntary participation with informed consent. Exclusion criteria included: (a) documented cancer recurrence; (b) prior mental disorders or intellectual disabilities; (c) psychological intervention received within 3 months before enrollment; (d) recent use of antidepressant or anti-anxiety drugs; and (e) critical illness precluding self-report completion. One-on-one questionnaires were distributed to eligible participants. Of 1,050 distributed, 979 were fully completed and returned, yielding a response rate of 92.3%.

### Questionnaire

A general questionnaire was used to collect demographic and clinical characteristics data. Demographic information (age, gender, occupation, marital status, education, and primary caregiver) was gathered via interview or self-report. Clinical data including tumor type, TNM stage, and radiotherapy history were extracted from the hospital’s electronic medical record system.

The 24-item DS-MV was adopted as the primary assessment tool, comprising five dimensions: loss of meaning (5 items), dysphoria (5 items), disheartenment (5 items), helplessness (4 items), and sense of failure (5 items). Rated on a 5-point Likert scale, it assesses participants’ psychological status over the prior 2 weeks via self-report. Consistent with prior research, the scale exhibited acceptable internal consistency in this sample: the overall Cronbach’s *α* was 0.901, with subscale coefficients of 0.810, 0.723, 0.826, 0.734, and 0.724 for loss of meaning, dysphoria, disheartenment, helplessness, and sense of failure, respectively.

### Data collection

Prior to formal investigation, a pilot test was conducted among four cancer patients to evaluate the questionnaire’s readability, clarity and applicability. The instructions and item expressions were then revised to improve clarity and feasibility. During formal data collection, uniformly trained researchers elaborated on the study objectives, procedures, significance and questionnaire guidelines for participants. After voluntary written informed consent was obtained, participants completed paper questionnaires with on-site verification; ambiguous responses were clarified immediately to ensure accurate and complete data collection. Data collection was arranged 1–2 days after admission to avoid disrupting patients’ routine clinical treatment.

### Data analysis

Prior to formal statistical analysis, raw data were first processed through missing data handling and outlier screening. Missing data were processed in two rigorous steps. The overall missing rate across all DS-MV items was 0.379%. Eight invalid questionnaires were excluded for participants with over 12.5% missing items (≥3 items). Little’s MCAR test confirmed remaining sporadic missing data were completely random (*p* > 0.05) and were subsequently imputed via multiple imputation. After data cleaning, 971 valid samples were included for final analysis, which far exceeded the minimum methodological requirement (150 patients required to estimate item difficulty within ±0.5 logits, *α* = 0.01, *β* = 0.2) (Linacre, 1994).

For outlier screening, DS-MV total Z-scores were calculated, identifying four extreme cases with Z > 3.29. Three researchers independently reviewed their complete medical records regarding disease stage, treatment history, and comorbid psychiatric conditions, and unanimously confirmed these cases reflected genuine extreme psychological states. Further batch and investigator tracking ruled out outlier clustering caused by non-standardized survey administration.

Statistical analyses were performed via IBM SPSS Statistics 27.0 and Winsteps 3.72.3. Classic test theory (CTT) analyses covering descriptive statistics, item discrimination, internal consistency and test–retest reliability were processed in SPSS, while Rasch analysis was conducted in Winsteps using the partial credit Rasch model (PCM).

Under the CTT framework, item discrimination was examined using Pearson’s correlation (≥0.30, *p* < 0.001) (DeVellis, 1991). Cronbach’s *α* was calculated for internal consistency (≥ 0.70 acceptable), with items removed if *α* increased by ≥0.02 after single-item deletion (DeVellis, 1991). Construct validity was assessed via Principal component analysis (KMO > 0.6, Bartlett’s test *p* < 0.001). Factors with eigenvalues >1 were extracted with the optimal quantity determined by parallel analysis and the scree plot. Following Promax oblique rotation (Fabrigar et al., 1999), items with |loadings| < 0.4 or problematic cross-loading (≥ 0.4 on two components with |difference| < 0.2) were eliminated (Lambert et al., 1991).

Rasch analysis was performed following standard psychometric guidelines (Bond and Fox, 2003). The 5-point response scale was condensed to 3 points to resolve disordered thresholds (Robinson et al., 2016). Global model fit was assessed by the Global Root-Mean-Square Residual (<0.40 ideal, <0.50 acceptable). Item and person fit were evaluated using fit residuals (ideal: mean near 0, SD ≤ 2.0; good fit: |Residual| ≤ 2.0; acceptable fit: |Residual| ≤ 3.0), INFIT MNSQ (ideal: 0.8–1.2; acceptable: 0.7–1.3), INFIT ZSTD (±2) and item/person fit residuals (SD ≤ 2.0) (Park and Park, 2019). Both the Rasch-derived Person Separation Index (PSI) and Cronbach’s α indicated acceptable reliability at a threshold of ≥0.70 (Santos, 1999). Gender- and age-based differential item functioning (DIF) was tested at a Bonferroni-adjusted α of 0.01 (Omara et al., 2025). Unidimensionality was verified when the first unexplained eigenvalue from residual PCA was <3.0. Local item dependency was identified with residual correlations |*r*| < 0.3 (Yen, 1984). Item targeting was assessed using person-item distribution graphs and collocated items were deleted based on item maps, Rasch fit statistics and face validity (Bond and Fox, 2003; DeVellis, 1991). The intraclass correlation coefficient (ICC) was used to examine test–retest reliability in a stable clinical subsample (two-way random-effects model; ICC > 0.75 indicated excellent reliability) (Weir, 2005).

### Ethical considerations

This study’s initial data collection was approved by the Ethics Committee of the Second Xiangya Hospital, Central South University (Approval No. LYG2022110). The ethics approval for the test–retest reliability assessment of the s-DS-MV was granted by the Ethics Committee of Xiangya School of Nursing, Central South University (Approval No. E202405). Before enrollment, all eligible participants received verbal and written explanations of the study aims, procedures and rights, and provided written informed consent. All procedures complied with the Declaration of Helsinki and the Ethical Guidelines for human clinical research.

## Results

### Sample characteristics

This study enrolled 971 participants aged 18–78 years (mean 53.97 ± 9.83 years). The cohort was predominantly female (85.0%, *n* = 825), with males accounting for the remaining 15.0% (*n* = 146). In terms of employment status, 313 participants (32.2%) were employed, while 658 (67.8%) were unemployed. Most participants were married (90.5%, *n* = 879), 19 (2.0%) were unmarried, and 73 (7.5%) were divorced or widowed. Regarding educational attainment, 262 participants (27.0%) had primary school education or below, 388 (40.0%) had junior high school education, 187 (19.3%) had senior high or vocational school education, and 134 (13.8%) held a college degree or above. Informal caregivers, defined as those providing daily care and emotional support to participants, were grouped by their relationship with patients: spouses (60.6%, *n* = 588), children (29.8%, *n* = 289), parents (3.3%, *n* = 32), and other relatives or non-relatives (6.4%, *n* = 62). Cancer types distribution was as follows: reproductive system cancers (78.4%, *n* = 761), digestive system cancers (8.8%, *n* = 85), thoracic cancers (6.3%, *n* = 61), head and neck cancers (4.5%, *n* = 44), urinary system cancers (1.8%, *n* = 17), and bone or soft tissue cancers (0.3%, *n* = 3). According to the TNM staging system, 424 participants (43.7%) were at stage II, 391 (40.3%) as stage III, and 156 (16.1%) at stage IV. Additionally, 606 participants (62.4%) received concurrent radiotherapy.

### Scale evaluation

#### Item discrimination analysis using Pearson’s correlations

Pearson’s correlation analysis revealed that all 24 items were positively and significantly correlated with the total DS-MV score, with coefficients ranging from 0.321 to 0.739 (*p* < 0.001; Supplementary File 1). All coefficients exceeded the conventional 0.30 threshold for acceptable item discrimination.

#### Internal consistency analysis via Cronbach’s *α* coefficient

The overall Cronbach’s α coefficient of the DS-MV scale was 0.901. When each item was deleted individually, the recalculated α coefficient ranged from 0.892 to 0.901. No item deletion led to an α increase of ≥ 0.02, indicating that removing any item would not improve the scale’s internal consistency.

#### Construct validity analysis via exploratory factor analysis

Exploratory factor analysis (EFA) was conducted with all 971 participants to explore the underlying factor structure and refine the item pool. Robust results from KMO and Bartlett’s tests (KMO = 0.906, χ^2^ = 9358.650, *p* < 0.001) confirmed the feasibility of factor analysis. Principal Component Analysis (PCA) initially extracted 5 components (eigenvalues>1), explaining 57.706% of total variance; however, parallel analysis and Cattell scree plot inspection (Supplementary File 1) identified a 2-factor solution as optimal, accounting for 42.246% of total variance. For this optimal 2-factor model, PCA was performed to extract common factors, followed by Promax optimal oblique rotation, which yielded an interpretable two-factor structure. The inter-component correlation *r* = 0.456 verified the appropriateness of oblique rotation. Dual item screening criteria were applied based on the Pattern Matrix: items with low factor loadings (absolute value<0.4 across all components) or items with cross-loadings (absolute value ≥0.4 on both components, with an absolute loading difference<0.2) (Lambert et al., 1991). Only one item was eliminated: Item 10 (“I feel guilty”), as its absolute factor loadings on both components (Component 1: 0.322; Component 2: 0.263) were both less than 0.4, meeting the low factor loading criterion. After item removal, the dimensional assignment of all remaining items was clarified. The inter-component correlation of the optimized two-factor model was *r* = 0.445, further confirming the suitability of oblique rotation for the present data. Component 1 encompassed Items 2, 3, 4, 5, 7, 8, 9, 11, 13, 15, 16, 18, 21, 22, 23, and 24; Component 2 included Items 1, 6, 12, 14, 17, 19, and 20 (Table 1).

**Table 1:** Pattern matrix of the revised DS-MV: two-component PCA with Promax oblique rotation.

**Item**	**Component 1**	**Component 2**
I can do many valuable things for others.	−0.283	0.779^a^
My life seems to be pointless	0.486^a^	0.297
There is no purpose to the activities in my life	0.476^a^	0.248
My role in life has been lost	0.495^a^	0.353
I no longer feel emotionally in control	0.535^a^	0.02
I am in good spirits	−0.146	0.705^a^
No one can help me	0.546^a^	0.038
I feel that I cannot help myself	0.607^a^	0.091
I feel hopeless	0.582^a^	0.254
**I feel guilty**	**0.322**	**0.263**
I feel irritable	0.754^a^	−0.064
I cope well with life	−0.002	0.496^a^
I have a lot of regret about my life	0.599^a^	−0.261
Life is no longer worth living	0.250	0.417^a^
I tend to feel hurt easily	0.758^a^	−0.231
I am angry about a lot of things	0.790^a^	−0.315
I am proud of what I have accomplished	−0.113	0.640^a^
I feel distressed about what is happening to me	0.657^a^	−0.151
I am a worthwhile person	−0.087	0.752^a^
I would rather not be alive	0.303	0.432^a^
I feel sad and miserable	0.720^a^	−0.012
I feel discouraged about life	0.625^a^	0.238
I feel quite isolated or alone	0.621^a^	0.147
I feel trapped by what is happening to me	0.696^a^	−0.063

Items 1, 6, 12, 17 and 19 were reverse-scored prior to data analysis to ensure consistent measurement of demoralization levels. Negative factor loadings indicate negative correlations between items and the connotations of their corresponding factors. Items marked with superscript a were retained following item screening. Bold values = factor loadings for eliminated Item 10 (|loadings| < 0.40 on both components).

### Rasch analysis

**Figure 1:**
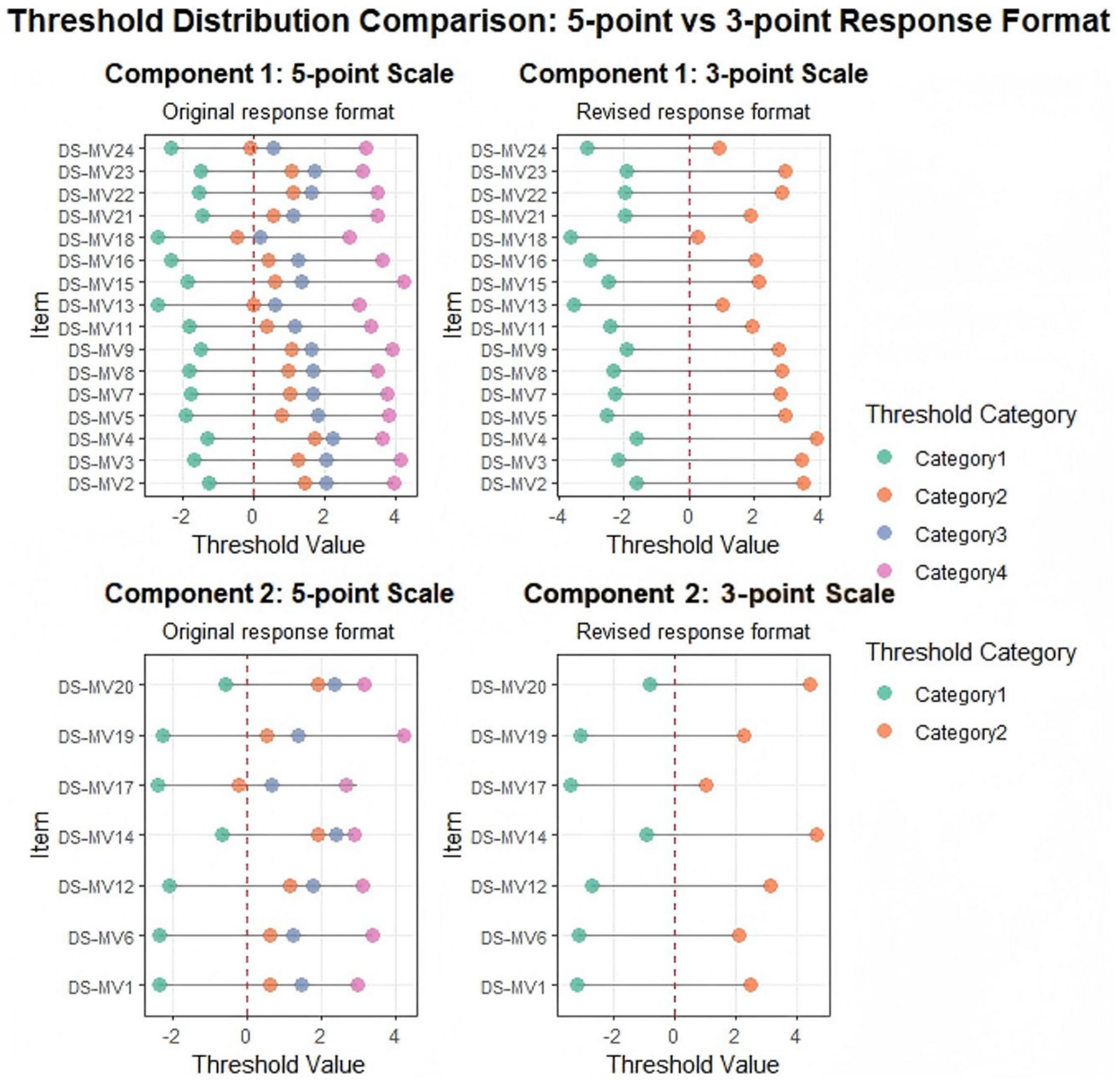
Item threshold distributions for the two factors of the DS-MV scale: Comparison of the original 5-point vs. revised 3-point response formats via PCM. Top: Component 1 (16 items); Bottom: Component 2 (7 items). Four colors (Category 1–4) in 5-point panels (left) and two colors (Category 1–2) in 3-point panels (right) represent scale thresholds.

The two components derived from PCA were each analyzed using the Partial Credit Model (PCM) in Winsteps. Under the original 5-point response format (0 = Never, 1 = Seldom, 2 = Sometimes, 3 = Often, 4 = All the time), disordered thresholds were detected for all items across both subscales, indicating suboptimal response category functioning. This finding corroborated our observations during scale administration: patients used adjacent response options inconsistently (i.e., *seldom* vs. *sometimes*, *often* vs. *all the time*) (Robinson et al., 2016; Yngve et al., 2018). To address this issue, we collapsed the 5-point response categories into three distinct options (0 = Never, 1 = Sometimes, 2 = Often). Repeating the PCM analysis with this revised format resolved all disordered thresholds for all items across both subscales (Figure 1). EAP reliability remained excellent for Component 1 (0.915 at 5-point and 0.904 at 3-point) and acceptable for Component 2 (0.762 at 5-point and 0.776 at 3-point), indicating no meaningful loss in reliability following the response category revision.

#### Subscale 1

Rasch analysis of this 16-item subscale (3-point scale: 0 = Never, 1 = Sometimes, 2 = Often) revealed acceptable overall model fit, with slight local misfit detected at the item level. For item fit, INFIT MNSQ ranged from 0.70 to 1.37, and INFIT ZSTD from −4.9 to 7.8. Four of the 16 items exhibited misfit beyond the acceptable criteria: item 13 (INFIT MNSQ = 1.37, ZSTD = 7.8), item 18 (INFIT MNSQ = 1.20, ZSTD = 5.1), item 22 (INFIT MNSQ = 0.76, ZSTD = -4.9), and item 4 (INFIT MNSQ = 0.78, ZSTD = -4.6). Notably, item 13 had an INFIT MNSQ marginally above the upper target limit of 1.3, whereas the other three items showed acceptable MNSQ values but deviant ZSTD estimates. For person fit, the analysis included 971 participants, among whom 22 had extreme scores. Excluding these extreme cases (*n* = 949), INFIT MNSQ ranged from 0.12 to 4.73, and INFIT ZSTD from −2.9 to 5.1. A small number of non-extreme participants presented severe misfit, with a maximum INFIT MNSQ of 4.73 and ZSTD of 5.1. Nevertheless, these misfitting participants and items accounted for a negligible proportion of the total sample, and did not impair the overall model fit. No differential item functioning (DIF) was found across gender and age groups (Supplementary File 1). Unidimensionality and local dependency were examined via principal component analysis (PCA) of standardized residuals (Table 2; Analysis 1). The Rasch model explained 43.4% of the raw variance, including 27.8% attributed to person ability and 15.5% to item difficulty. The first unexplained contrast yielded an eigenvalue of 2.4 (< 3.0), accounting for only 14.8% of the unexplained variance, with subsequent eigenvalues showing a monotonic decline. No evidence of local dependency was identified.

**Table 2:** Summary of Rasch model fit statistics for the scales.

**Scale**	**Analysis**	**GRMS** ^‡^	**Item fit residual** ^†^	**Person fit residual** ^‡^	**Item INFIT MNSQ** ^†^	**Person INFIT MNSQ** ^‡^	**Person separation reliability** ^†^	**α** ^†^
Subscale 1								
16 items	1	0.428	−0.2 ± 3.3	−0.3 ± 1.9	0.98 ± 0.15	0.97 ± 0.72	0.90	0.909
9 items (items 4, 7, 8, 13, 18, 22 and 23 removed)	2	0.418	−0.3 ± 2.0	−0.2 ± 1.5	0.99 ± 0.10	0.94 ± 0.85	0.84	0.855
Subscale 2								
7 items	3	0.382	−1.3 ± 1.9	−0.3 ± 1.3	0.94 ± 0.09	0.93 ± 0.96	0.80	0.775
5 items (item 17, 19 removed)	4	0.357	−1.4 ± 1.3	−0.3 ± 1.2	0.94 ± 0.10	0.93 ± 1.00	0.74	0.708
Total: 14 items	5	0.436	−0.3 ± 2.9	−0.2 ± 1.7	0.98 ± 0.14	0.97 ± 0.70	0.86	0.864

Non-extreme participants by analysis: Analysis 1 (*n* = 949); Analysis 2 (*n* = 939); Analysis 3 (*n* = 958); Analysis 4 (*n* = 943); Analysis 5 (*n* = 962). Symbol definitions: † = full sample (*n* = 971); ‡ = non-extreme participants. Fit criteria: Global Root-Mean-Square Residual (GRMS; also called GRMR) assessed overall model–data fit (ideal: <0.4; acceptable: <0.50). Item/person fit residuals: ideal (mean ≈ 0, SD ≤ 2.0), good (|Residual| ≤ 2.0), acceptable (|Residual| ≤ 3.0). INFIT MNSQ: ideal (0.8–1.2), acceptable (0.7–1.3). Person Separation Reliability (equivalent to Person Separation Index, PSI) evaluated person discrimination: ≥0.70 (acceptable; PSI ≈ 1.53), ≥0.80 (good; PSI = 2.0), ≥0.90 (excellent; PSI ≈ 2.58). α (Cronbach’s α) indexed internal consistency: ≥0.70 (acceptable), ≥0.80 (good), ≥0.90 (excellent).

#### Subscale 2

Rasch analysis of this 7-item subscale (3-point scale) yielded acceptable overall model fit. For item fit, INFIT MNSQ ranged from 0.82 to 1.10 and INFIT ZSTD from −3.8 to 2.2. Two items exhibited misfit: item 14 (INFIT MNSQ = 0.83, ZSTD = -3.5) and item 19 (INFIT MNSQ = 0.82, ZSTD = -3.8). For person fit, the analysis included 971 participants, among whom 13 had extreme scores. Excluding these extreme cases (n = 958), INFIT MNSQ ranged from 0.17 to 9.43 and INFIT ZSTD from −1.5 to 5.3. A small subset of non-extreme participants presented extreme misfit, with a maximum INFIT MNSQ of 9.43 and ZSTD of 5.3. Nonetheless, these misfitting cases accounted for a negligible proportion of the total sample and did not impair the overall model fit. No DIF was detected across age groups. By contrast, Item 12 (“I cope well with life”) displayed uniform DIF across gender (Bonferroni-adjusted *p* = 0.008 < 0.01; Supplementary File 1). The Rasch model explained 52.1% of the raw variance, comprising 36.4% for person ability and 15.7% for item difficulty. The first unexplained contrast yielded an eigenvalue of 1.8 (<3.0), with subsequent eigenvalues declining monotonically to ≤1.5. Unidimensionality and local independence were examined via PCA of standardized residuals (Table 2; Analysis 3), with no evidence of local dependency identified.

### Scale modification

Based on item map inspection of item colocation within each subscale, Items 4, 7, 8, 13, 18, 22 and 23 were removed from Subscale 1. The retained items were defined as the Existential Emptiness and Affective Distress dimension. Meanwhile, Items 17 and 19 were excluded from Subscale 2, with the remaining items labeled as the Self-Worth and Life Valuation dimension.

#### Existential emptiness and affective distress

The revised 9-item subscale achieved adequate Rasch model fit: item fit residual (mean ± SD) = −0.3 ± 2.0 and person fit residual (mean ± SD) = −0.2 ± 1.5, both within the target range (SD ≤ 2.0). Two items presented marginal misfit with |INFIT ZSTD| ranging from 2.2 to 2.6, which remained within acceptable limits, and no extreme outliers were identified. The overall model log-likelihood chi-square was statistically significant (*χ^2^* = 9437.79, df = 7,503, *p* < 0.001), supporting satisfactory model fit.

No DIF was detected across gender and age groups. Unidimensionality was verified, with detailed results shown in Table 2 (Analysis 2). The Rasch model accounted for 43.6% of the raw variance, including 32.6% attributed to person ability and 11.0% to item difficulty. The eigenvalue of the first unexplained contrast was 1.9, lower than the value of 2.4 in the original 16-item version. Subsequent eigenvalues declined monotonically (1.5, 1.2, 1.1, 0.9), all falling below 1.5.

**Figure 2:**
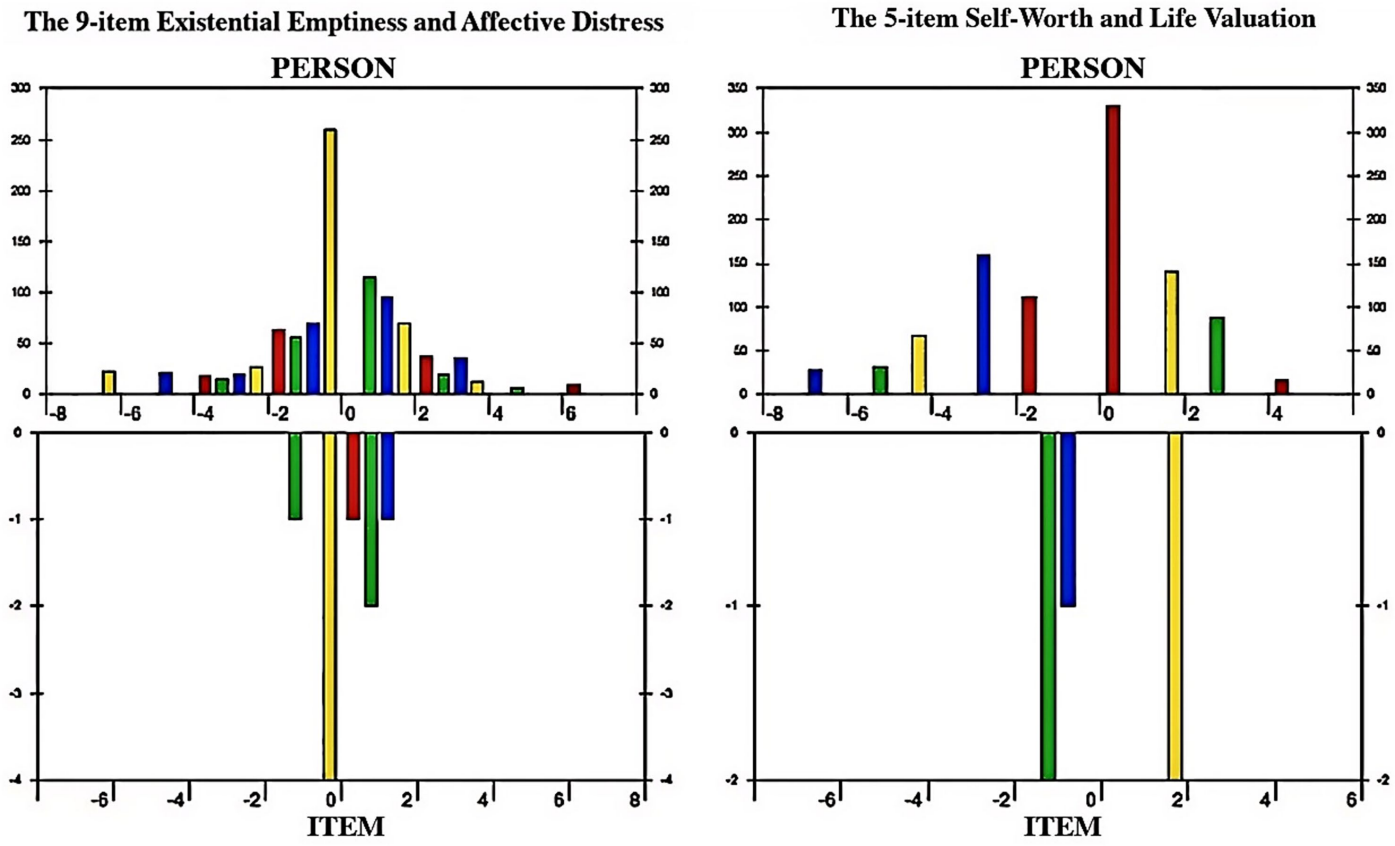
Person-item threshold distribution of the revised subscales derived from Rasch analysis.

No local item dependency was found; the maximum standardized residual correlation was 0.24 (between Item 2 and Item 3). Overall, the subscale showed adequate targeting: items were distributed across a core difficulty range of −1.41 to 1.03 logits, adequately capturing respondents’ trait distribution (Figure 2), albeit with limited coverage for extremely high and low trait levels.

#### Self-worth and life valuation

The revised 5-item subscale achieved adequate Rasch model fit: item fit residual (mean ± SD) = −1.4 ± 1.3 and person fit residual (mean ± SD) = −0.3 ± 1.2, both within the target range (SD ≤ 2.0). The overall model log-likelihood chi-square was statistically significant (*χ^2^* = 4200.57, df = 3,767, *p* < 0.001), supporting satisfactory model fit. All items showed well-calibrated psychometric properties, with INFIT MNSQ ranging from 0.82 to 1.03 and OUTFIT MNSQ from 0.95 to 1.15. No extreme respondent outliers were identified.

No DIF was detected across age groups. Only Item 12 (“I cope well with life”) exhibited uniform DIF across gender (Bonferroni-adjusted *p* = 0.005 < 0.01). Unidimensionality was verified, with detailed results presented in Table 2 (Analysis 4). The Rasch model accounted for 53.9% of the raw variance, including 42.3% attributed to person ability and 11.6% to item difficulty. The first unexplained contrast yielded an eigenvalue of 1.8, with subsequent eigenvalues declining monotonically (1.2, 1.0, 0.9, 0.1) and all falling below 1.2.

No severe local item dependency was found. The maximum standardized residual correlation was −0.38 (between item 1 and item 20; 0.3 < |*r*| < 0.4). Overall, the subscale demonstrated adequate targeting: items were distributed across a core difficulty range of −1.49 to 1.71 logits, adequately capturing respondents’ traits distribution (Figure 2), albeit with limited coverage for extremely high or low trait levels.

### Total scale

To verify the appropriateness of aggregating subscale scores to generate a total score representing the latent construct of demoralization, we conducted a subtest analysis (Table 2; Analysis 5). Results supported the unidimensionality of the underlying demoralization construct. The original 24-item DS-MV was refined into the final 14-item s-DS-MV through Rasch analyses, with response options collapsed to a 3-point ordinal scale. Two subscales were derived: Existential Emptiness and Affective Distress (9 items) and Self-Worth and Life Valuation (4 items). Subscale scores were calculated as the sum of their respective items, and a total demoralization score was computed from all 14 items, with higher scores indicating more severe demoralization.

Internal consistency coefficients for the total s-DS-MV and its subscales are reported in Table 2. Among a subsample of clinically stable patients recruited in September 2024 (*n* = 50; test–retest interval = 4.54 ± 0.676 days), the s-DS-MV was administered twice, demonstrating excellent test–retest reliability. The intraclass correlation coefficient (ICC) for the total s-DS-MV was 0.933 [95% CI = 0.885–0.962], with corresponding ICC values of 0.934 [95% CI = 0.886–0.962] for the Existential Emptiness and Affective Distress subscale and 0.881 [95% CI = 0.799–0.931] for the Self-Worth and Life Valuation subscale. The Spearman correlation between the two subscales was 0.486 (*p* < 0.001), supporting that both subscales tap into the common construct of demoralization while justifying their distinct dimensional structure.

The total scale and both subscales were negatively skewed, with scores concentrated around each scale’s mid-range: medians were exactly aligned with their respective theoretical midpoints, and 50% of scores fell within a narrow interquartile range. Whereas the Existential Emptiness and Affective Distress subscale spanned its full possible range, the total scale and the Self-Worth and Life Valuation subscale did not reach their theoretical upper limits, indicating a mild ceiling effect limited to these two scales. As shown in Table 3, the observed score distributions confirmed that most participants reported moderate levels of demoralization, with a slight concentration toward higher severity consistent with the negative skew.

**Table 3:** Descriptive Statistics for the s-DS-MV.

Variable	s-DS-MV	Existential emptiness and affective distress	Self-worth and life valuation
Mean ± SD	13.48 ± 4.59	9.03 ± 3.46	4.45 ± 1.73
Median	14	9	5
Interquartile range (P25, P75)	5 (11, 16)	3 (8, 11)	3 (3, 6)
Observed range	0–24	0–18	0–8
Possible range	0–28	0–18	0–10
Skewness	−0.732	−0.370	−0.499
Kurtosis	0.418	0.726	−0.030

SD = standard deviation.

The s-DS-MV exhibited a mild ceiling effect at the highest severity levels, which limits its ability to discriminate among individuals with very high demoralization. Nevertheless, scores clustered tightly around each scale’ s mid-range, indicating strong performance in assessing low to moderate levels of demoralization — rendering it well-suited for clinical screening purposes. The final version of the s-DS-MV is presented in Table 4.

**Table 4:** Items and scoring of the 14-item short-form demoralization scale-mainland version (s-DS-MV).

**Item no.**	**Item content**	**Never**	**Sometimes**	**Often**
1^¶^	I can do many valuable things for others.	0	1	2
2	My life seems to be pointless.	0	1	2
3	There is no purpose to the activities in my life.	0	1	2
4	I no longer feel emotionally in control.	0	1	2
5^¶^	I am in good spirits.	0	1	2
6	I feel hopeless.	0	1	2
7	I feel irritable.	0	1	2
8^¶^	I cope well with life.	0	1	2
9	Life is no longer worth living.	0	1	2
10	I tend to feel hurt easily.	0	1	2
11	I am angry about a lot of things.	0	1	2
12	I would rather not be alive.	0	1	2
13	I feel sad and miserable.	0	1	2
14	I feel trapped by what is happening to me.	0	1	2

For each statement, select the option that best reflects your feelings over the past 2 weeks. Scoring instructions: Total demoralization score: sum of scores for all 14 items; Existential Emptiness and Affective Distress subscale: sum of scores for Items 2, 3, 4, 6, 7, 10, 11, 13, and 14; Self-Worth and Life Valuation subscale: sum of scores for Items 1, 5, 8, 9, and 12. ^¶^ Reverse-scored item (reverse coding required prior to total/subscale score calculation).

## Discussion

In this study, we conducted comprehensive psychometric validation and targeted refinement of the Mandarin version of the Demoralization Scale (DS-MV), leveraging an integrated analytical framework of classical test theory (CTT) and item response theory (IRT). To avoid losing core demoralization constructs, all retained and excluded items were systematically mapped against the original 24-item DS-MV framework; as shown in Supplementary Table 4, items were retained across all five core dimensions (disheartenment, dysphoria, helplessness, loss of meaning, and sense of failure), fully preserving the key conceptual domains and theoretical structure of demoralization. Our work yielded the final 14-item two-dimensional short-form Mandarin version of the Demoralization Scale (s-DS-MV), which preserves the full conceptual framework of demoralization (Fang et al., 2014), reduces the original item pool from 24 to 14 items, markedly reduces patient response burden, and retains robust psychometric properties with strong clinical utility.

In this study, we first determined the most appropriate structural framework for the scale. The optimal two-factor structure was identified via exploratory factor analysis, parallel analysis, and scree plot inspection, with the appropriateness of oblique rotation verified by an inter-factor correlation of *r* = 0.445. The final s-DS-MV consists of two theoretically aligned, conceptually distinct subscales: the 9-item Existential Emptiness and Affective Distress subscale, which captures core multidimensional content from the original scale and maps primarily onto the affective-experiential domain of demoralization; and the 5-item Self-Worth and Life Valuation subscale, which integrates items tapping into perceptions of failure and loss of meaning (including reverse-scored items) to assess self-worth, coping capacity, meaning in life, and will to live, corresponding to the existential-cognitive dimension of demoralization. This two-factor structure was refined via factor loading screening, Rasch fit evaluation, local dependency testing, and clinical relevance review, demonstrating robust structural validity and stability. The two subscales were moderately correlated (Spearman *r* = 0.486, *p* < 0.001), with only 23.6% shared variance. This pattern indicates that the subscales measure distinct yet interrelated facets of the overarching demoralization construct, providing strong support for the structural integrity and discriminant validity of our two-factor model.

Guided by the dual priorities of psychometric rigor and clinical applicability, we completed item reduction and response format optimization in a standardized, evidence-based manner. We sequentially excluded misfitting and redundant items based on prespecified criteria, including factor loadings, item fit statistics, local dependency, and differential item functioning (DIF), while retaining the scale’s core measurement structure. In parallel, we systematically revised the original scale’s response format to address observed response barriers: with the original 5-point rating scale, we identified substantial threshold disordering across all items, indicating that patients could not reliably differentiate between adjacent response options. After collapsing the scale to a simplified 3-point scoring system (0 = Never, 1 = Sometimes, 2 = Often), all threshold disordering was fully resolved, with substantial improvements in measurement precision and no compromise to scale reliability. These findings confirm that this streamlined response format is better suited for patients with advanced cancer and limited cognitive reserve (Robinson et al., 2016).

We further performed in-depth psychometric validation using the Rasch model to confirm the scale’s measurement performance. First, unidimensionality was verified for both subscales via principal component analysis of standardized residuals; eigenvalues for the first unexplained contrast were all below 3.0, fully satisfying the core unidimensionality assumptions of the Rasch model. Second, post-refinement item fit indices (INFIT MNSQ, OUTFIT MNSQ) were globally within the widely accepted range of 0.7–1.3, with most falling within the ideal 0.8–1.2 bandwidth, and only negligible marginal misfit observed. Third, DIF analyses across gender and age groups revealed that nearly all items exhibited no meaningful measurement bias. Only Item 12 (“I cope well with life”) showed uniform gender DIF, whose statistical significance may be partly attributed to the large sample size and imbalanced gender distribution. The DIF magnitude was 0.80 logits, below the 1.0 logit cutoff for clinically substantive bias, and this uniform DIF only caused a stable item difficulty shift between genders without changing respondents’ relative ordering on the latent trait. Importantly, we retained Item 12 based on a comprehensive integration of statistical performance, conceptual significance, and clinical relevance, rather than purely statistical criteria. This item is a core indicator of demoralization in advanced cancer patients and directly supports clinical coping assessment and the development of subsequent targeted psychological interventions, justifying its retention. Finally, person-item targeting analyses demonstrated that item difficulty clustered within the core score range, enabling robust discrimination of mild-to-moderate demoralization. Although coverage was limited for extreme symptom severity, this profile aligns closely with the primary goals of routine clinical screening.

Notably, the s-DS-MV exhibits a mild ceiling effect at the highest severity levels of demoralization, which may limit its ability to discriminate among patients with extremely severe symptoms. Despite this limitation, the scale performs exceptionally well in assessing low-to-moderate levels of demoralization, as scores are densely concentrated around the mid-range of each subscale. This pattern aligns perfectly with the NCCN Guidelines’ recommendation for routine distress monitoring in oncology practice (NCCN, 2026), making the s-DS-MV highly suitable for rapid, repeated screening and timely targeted intervention—its core clinical purpose. Furthermore, this skewed score distribution and mild ceiling effect are consistent with the real-world symptom distribution among cancer patients (Fang et al., 2014; Hong et al., 2022; Li et al., 2025).

With respect to reliability, the full s-DS-MV and its subscales adhered to well-established psychometric benchmarks for clinical measurement, demonstrating satisfactory internal consistency and favorable performance on the item response theory (IRT)-derived person separation index (PSI). In a subsample of 50 clinically stable patients, intraclass correlation coefficients (ICCs) for both the full scale and subscales all exceeded 0.88, indicating excellent test–retest reliability. This finding further validates the clinical utility of the s-DS-MV for repeated symptom assessment and longitudinal monitoring of demoralization.

### Limitation

This study has several limitations. First, mild negative skewness and a high-end ceiling effect limited discrimination of extreme demoralization, though this distribution matches the real symptom profile of clinical cancer patients. Second, this study adopted convenience sampling from two tertiary hospitals in Hunan Province, which recruit both local and cross-regional patients. However, the sample had a marked gender imbalance, with females accounting for 85% of all participants, which may introduce potential gender bias and limit the scale’s psychometric performance and external generalizability. We therefore conducted DIF analyses across gender and age groups; results showed negligible measurement bias for most items, and only Item 12 exhibited uniform gender DIF. After comprehensive assessment of its clinical connotation and overall psychometric properties, Item 12 was ultimately retained. We acknowledge that such isolated item-level DIF may slightly compromise strict statistical gender measurement invariance, and further validation in more gender-balanced clinical cohorts is recommended to verify and optimize the performance of Item 12 in future research. Even though the participating hospitals enroll patients from neighboring regions, nationwide generalization of the scale is not warranted. The scale is currently primarily applicable to advanced cancer patients with comparable demographic and clinical characteristics in Hunan Province and its surrounding areas, and caution is advised when extending its application to other populations before further cross-regional validation. Third, the stability of the two-factor structure needs further verification via confirmatory factor analysis based on a new independent clinical sample, which is a critical priority for our future clinical research. Finally, as participants were mainly advanced cancer patients, the results cannot be arbitrarily generalized to palliative care patients or patients with other chronic diseases.

## Conclusion

In summary, the 14-item s-DS-MV is concise, user-friendly, and psychometrically robust, ideal for rapid screening and longitudinal monitoring of demoralization among advanced cancer patients with similar profiles in Hunan and adjacent regions. Although developed in large tertiary hospitals treating local and cross-provincial patients, the scale requires cautious nationwide application and has no direct cross-cultural applicability. It nonetheless provides methodological references for demoralization assessment nationwide and can serve as a tool for routine psychological evaluation and intervention outcome monitoring to support standardized clinical demoralization management.

## Data Availability

The raw data supporting the conclusions of this article will be made available by the authors, without undue reservation.
