# Supplementary material for: Development and psychometric validation of the short-form mandarin Chinese demoralization scale for cancer patients
Source: Front Psychol. 2026 Jun 16;17:1834425. doi: 10.3389/fpsyg.2026.1834425 (PMC13314784; doi:10.3389/fpsyg.2026.1834425)
Supplement: Supplementary file 1 [file Supplementary_file_1.DOCX]

## **Supplementary Figure 1. Parallel Analysis for DS-MV Scale**
