# Supplementary figures and images for: Development and psychometric validation of the short-form mandarin Chinese demoralization scale for cancer patients
Source: Front Psychol. 2026 Jun 16;17:1834425. doi: 10.3389/fpsyg.2026.1834425 (PMC13314784; doi:10.3389/fpsyg.2026.1834425)

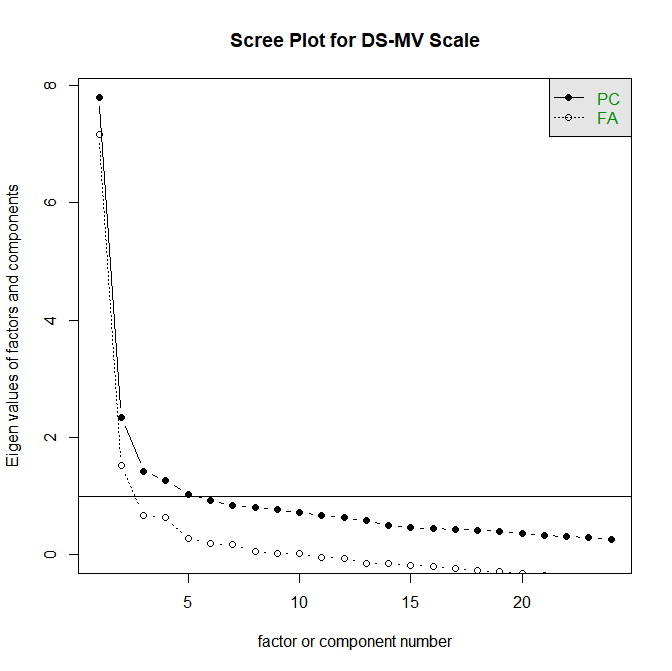


## **Supplementary Figure 2. Scree Plot for DS-MV Scale**

Supplement: Supplementary file 2 [file Supplementary_file_2.DOCX]
