# Supplementary material for: Development and psychometric validation of the short-form mandarin Chinese demoralization scale for cancer patients
Source: Front Psychol. 2026 Jun 16;17:1834425. doi: 10.3389/fpsyg.2026.1834425 (PMC13314784; doi:10.3389/fpsyg.2026.1834425)
Supplement: Supplementary file 3 [file Supplementary_file_3.DOCX]

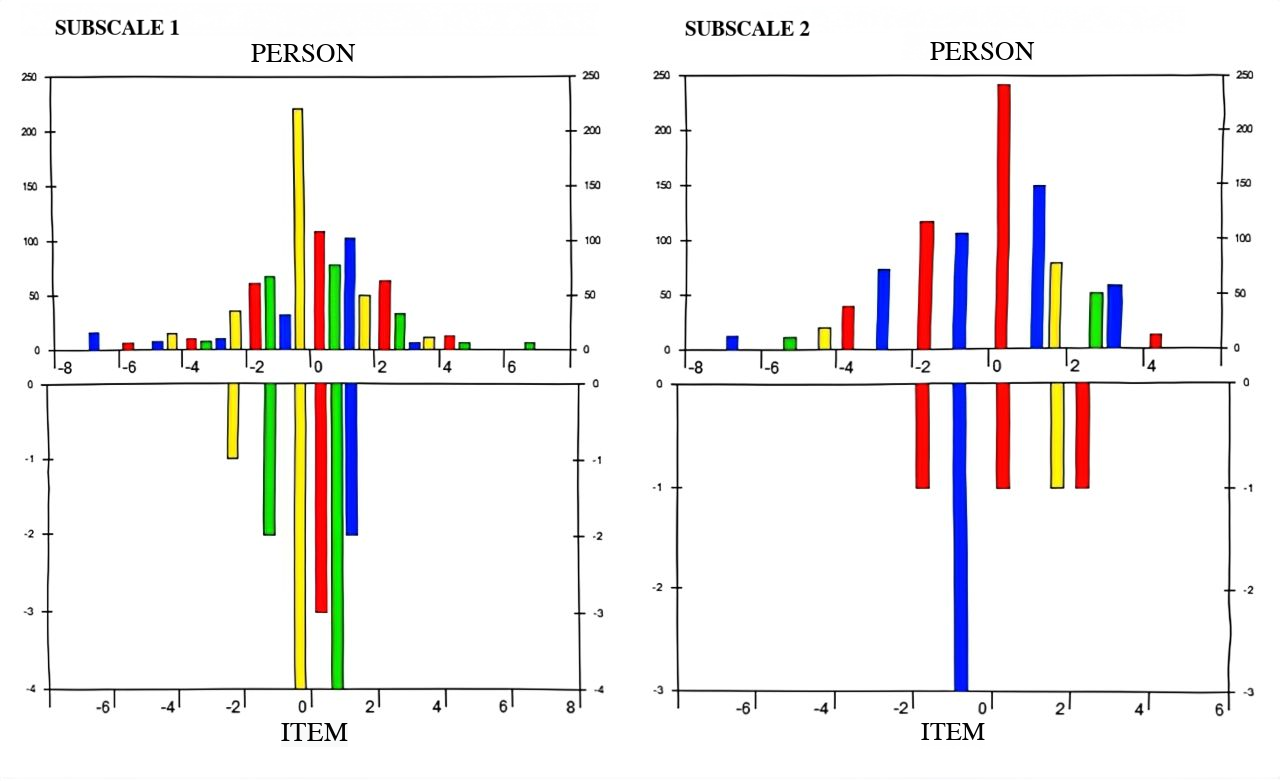


## **Supplementary Figure 3. Person-item threshold distribution of the original subscales derived from Rasch analysis**
