# Supplementary material for: Development and psychometric validation of the short-form mandarin Chinese demoralization scale for cancer patients
Source: Front Psychol. 2026 Jun 16;17:1834425. doi: 10.3389/fpsyg.2026.1834425 (PMC13314784; doi:10.3389/fpsyg.2026.1834425)
Supplement: Supplementary file 4 [file Table_1.docx]

## Supplementary Table 1. Pearson Correlation Coefficients Between Each Item and Total DS-MV Score

| **Item** | ***r*** | **Item** | ***r*** | **Item** | ***r*** | **Item** | ***r*** | **Item** | ***r*** | **Item** | ***r*** |
| --- | --- | --- | --- | --- | --- | --- | --- | --- | --- | --- | --- |
| DS-MV01 | .321^**^ | DS-MV13 | .433^**^ | DS-MV05 | .526^**^ | DS-MV20 | .557^**^ | DS-MV24 | .634^**^ | DS-MV21 | .671^**^ |
| DS-MV12 | .371^**^ | DS-MV19 | .472^**^ | DS-MV16 | .546^**^ | DS-MV15 | .576^**^ | DS-MV02 | .639^**^ | DS-MV04 | .680^**^ |
| DS-MV17 | .383^**^ | DS-MV10 | .479^**^ | DS-MV07 | .550^**^ | DS-MV03 | .602^**^ | DS-MV23 | .670^**^ | DS-MV09 | .718^**^ |
| DS-MV06 | .388^**^ | DS-MV14 | .501^**^ | DS-MV18 | .555^**^ | DS-MV08 | .632^**^ | DS-MV11 | .671^**^ | DS-MV22 | .739^**^ |

**Notes**:

1. ^**^ indicates statistical significance at the .001 level (two-tailed).

2. Items are sorted in ascending order of correlation coefficients with the total DS-MV score.
