# Supplementary material for: Development and psychometric validation of the short-form mandarin Chinese demoralization scale for cancer patients
Source: Front Psychol. 2026 Jun 16;17:1834425. doi: 10.3389/fpsyg.2026.1834425 (PMC13314784; doi:10.3389/fpsyg.2026.1834425)
Supplement: Supplementary file 5 [file Table_2.docx]

## **Supplementary Table 3. Sex and Age Fairness Evaluation via DIF for Items in Components 1 and 2**

| **Item No.** | **Sex** | | **Age^#^** | |
| --- | --- | --- | --- | --- |
|  | **DIF Contrast** | **Absolute Welch t-Value** | **DIF Contrast** | **Absolute Welch t-Value** |
| **Component 1** | | | | |
| 2 | -.33 | 1.6 | .08 | .46 |
| 3 | .21 | .96 | .00 | .00 |
| 4 | -.08 | .36 | .15 | .88 |
| 5 | -.29 | 1.34 | -.18 | 1.02 |
| 7 | .05 | .25 | .09 | .52 |
| 8 | -.06 | .30 | .22 | 1.22 |
| 9 | .00 | .00 | .10 | .54 |
| 11 | .39 | 1.83 | -.16 | .92 |
| **13** | **.64** | **3.16**^i^ | .00 | .00 |
| 15 | .38 | 1.77 | -.09 | .52 |
| 16 | .07 | .34 | .11 | .62 |
| 18 | -.08 | .39 | -.03 | .19 |
| 21 | -.30 | 1.4 | .00 | .00 |
| 22 | -.23 | 1.07 | -.22 | 1.26 |
| 23 | .02 | .10 | .05 | .28 |
| 24 | -.40 | 1.94 | -.04 | .25 |
| **Component 2** | | | | |
| 1 | -.41 | 1.7 | .03 | .17 |
| 6 | -.16 | .68 | .09 | .48 |
| **12** | **.80** | **3.29^ii^** | **.56** | **3.01^iii^** |
| 14 | -.25 | 1.14 | .00 | .00 |
| 17 | -.22 | .94 | -.41 | 2.23 |
| 19 | .24 | .99 | -.09 | .47 |
| 20 | .05 | .21 | -.2 | 1.08 |

**Notes:**

1. DIF = Differential Item Functioning.
2. Significant DIF (unfair) is defined only when both criteria are met: (1) Absolute DIF contrast > 0.5 logits; (2) Absolute Welch’s t-value > 1.96.
3. Group coding: Age – Non-elderly ( < 60 years = 0), Elderly ( ≥ 60 years = 1); Gender – Female = 0, Male = 1.
4. ^i^ Item 13 (“I have a lot of regret about my life”): Bonferroni-adjusted *p* = .029, greater than the corrected *α* level of .01, indicating no statistically significant DIF; ^ii^ Item 12 (“I cope well with life”): Bonferroni-adjusted *p* = .008, lower than the corrected *α* level of .01, presenting significant DIF; ^iii^ Item 12 (“I cope well with life”): Bonferroni-adjusted *p* = .019, exceeding the corrected *α* level of .01, with no significant DIF.
