# Supplementary material for: Development and psychometric validation of the short-form mandarin Chinese demoralization scale for cancer patients
Source: Front Psychol. 2026 Jun 16;17:1834425. doi: 10.3389/fpsyg.2026.1834425 (PMC13314784; doi:10.3389/fpsyg.2026.1834425)
Supplement: Supplementary file 6 [file Table_3.docx]

## **Supplementary Table 2. Cronbach’s Alpha if Item Deleted for Each Item of the DS-MV**

| **Item** | ***Cronbach’s Alpha if Item Deleted*** | **Item** | ***Cronbach’s Alpha if Item Deleted*** | **Item** | ***Cronbach’s Alpha if Item Deleted*** |
| --- | --- | --- | --- | --- | --- |
| DS-MV01 | .902 | DS-MV09 | .892 | DS-MV17 | .901 |
| DS-MV02 | .895 | DS-MV10 | .898 | DS-MV18 | .897 |
| DS-MV03 | .895 | DS-MV11 | .894 | DS-MV19 | .898 |
| DS-MV04 | .894 | DS-MV12 | .900 | DS-MV20 | .897 |
| DS-MV05 | .897 | DS-MV13 | .900 | DS-MV21 | .894 |
| DS-MV06 | .901 | DS-MV14 | .898 | DS-MV22 | .892 |
| DS-MV07 | .897 | DS-MV15 | .896 | DS-MV23 | .894 |
| DS-MV08 | .895 | DS-MV16 | .897 | DS-MV24 | .895 |
