# Supplementary material for: Development and psychometric validation of the short-form mandarin Chinese demoralization scale for cancer patients
Source: Front Psychol. 2026 Jun 16;17:1834425. doi: 10.3389/fpsyg.2026.1834425 (PMC13314784; doi:10.3389/fpsyg.2026.1834425)
Supplement: Supplementary file 7 [file Table_4.docx]

**Supplementary Table 4. Original 24-item DS-MV Items: Dimensional Affiliation and Reduction Status**

| **No.** | **Item Content** | **Dimensions** | **Rationale for Deletion** |
| --- | --- | --- | --- |
| ~~18~~ | ~~I feel distressed about what is happening to me~~ | ~~disheartenment~~ | INFIT ZSTD = 5.1 |
| 21 | I feel sad and miserable | disheartenment | - |
| ~~22~~ | ~~I feel discouraged about life~~ | ~~disheartenment~~ | INFIT ZSTD = -4.9 |
| ~~23~~ | ~~I feel quite isolated or alone~~ | ~~disheartenment~~ | Item map colocation and semantic redundancy |
| 24 | I feel trapped by what is happening to me | disheartenment | - |
| ~~10~~ | ~~I feel guilty~~ | ~~dysphoria~~ | Factor loading = 0.322 |
| 11 | I feel irritable | dysphoria | - |
| ~~13~~ | ~~I have a lot of regret about my life~~ | ~~dysphoria~~ | INFIT MNSQ = 1.37，INFIT ZSTD = 7.8 |
| 15 | I tend to feel hurt easily | dysphoria | - |
| 16 | I am angry about a lot of things | dysphoria | - |
| 5 | I no longer feel emotionally in control | helplessness | - |
| 7 | ~~No one can help me~~ | ~~helplessness~~ | Item map colocation and semantic redundancy |
| ~~8~~ | ~~I feel that I cannot help myself~~ | ~~helplessness~~ | Item map colocation and semantic redundancy |
| 9 | I feel hopeless | helplessness | - |
| 2 | My life seems to be pointless | loss of meaning | - |
| 3 | There is no purpose to the activities in my life | loss of meaning | - |
| ~~4~~ | ~~My role in life has been lost~~ | ~~loss of meaning~~ | INFIT ZSTD = -4.6 |
| 14 | Life is no longer worth living | loss of meaning | - |
| 20 | I would rather not be alive | loss of meaning | - |
| 1 | I can do many valuable things for others. | sense of failure | - |
| 6 | I am in good spirits | sense of failure | - |
| 12 | I cope well with life | sense of failure | - |
| ~~17~~ | ~~I am proud of what I have accomplished~~ | ~~sense of failure~~ | Item map colocation and semantic redundancy |
| ~~19~~ | ~~I am a worthwhile person~~ | ~~sense of failure~~ | INFIT ZSTD = -3.8 |
